# Case Report: Human Umbilical Cord Mesenchymal Stem Cells as a Therapeutic Intervention for a Critically Ill COVID-19 Patient

**DOI:** 10.3389/fmed.2021.691329

**Published:** 2021-07-08

**Authors:** Quan Zhang, Kang Huang, Jianlei Lv, Xiang Fang, Jun He, Ailian Lv, Xuan Sun, Lamei Cheng, Yanjun Zhong, Shangjie Wu, Yao Dai

**Affiliations:** ^1^Department of Pulmonary and Critical Care Medicine, The Second Xiangya Hospital, Central South University, Changsha, China; ^2^Department of Critical Care Medicine, First Hospital of Changsha, Changsha, China; ^3^School of Basic Medical Sciences, Institute of Reproductive and Stem Cell Engineering, Central South University, Changsha, China; ^4^National Engineering and Research Center of Human Stem Cells, Changsha, China; ^5^Department of Critical Care Medicine, The Second Xiangya Hospital, Central South University, Changsha, China

**Keywords:** COVID-19, mesenchymal stem cells, critically ill, therapy, case report

## Abstract

Here we report a critically ill patient who was cured of SARS-CoV-2 infection in Changsha, China. A 66-year-old Chinese woman, with no significant past medical history, developed severe pneumonia-like symptoms and later diagnosed as severe COVID-19 pneumonia. Within 2 months of hospitalization, the patient deteriorated to ARDS including pulmonary edema and SIRS with septic shock. When treatment schemes such as antibiotics plus corticosteroids showed diminished therapeutic value, hUCMSC therapy was compassionately prescribed under the patient's consent of participation. After treatment, there was significant improvement in disease inflammation-related indicators such as IL-4, IL-6, and IL-10. Eventually, it confirmed the therapeutic value that hUCMSCs could dampen the cytokine storm in the critically ill COVID-19 patient and modulated the NK cells. In the continued hUCMSC treatment, gratifying results were achieved in the follow-up of the patient. The data we acquired anticipate a significant therapeutic value of MSC treatment in severe and critically ill patients with COVID-19, while further studies are needed.

## Introduction

At the end of 2019, several cases of severe respiratory infections were identified in Wuhan, China, which were triggered by a novel virus that was later assigned the name SARS-CoV-2 or COVID-19. Individuals of all ages are vulnerable to COVID-19 infection; however, those aged over 60 and have preexisting medical conditions, such as cardiovascular disease, diabetes, or high blood pressure, are prone to become critically ill and exhibit higher risk of suffering from the condition. According to the Centers for Disease Control and Prevention, the symptoms of COVID-19, in some cases, include fever, cough, shortness of breath, fatigue, and sudden loss of sense of smell and taste, while corresponding typical characteristics are pneumonia that requires hospitalization and even death, in case of absence of timely intervention. Anti-inflammatory treatment has been proposed and implemented but challenged with the dilemma of balancing the risk of secondary infection.

This newly emerging virus infection pandemic may lead to multiple organ damage, and there is, so far, still lack of specific treatments or available drugs. It is especially suitable for stem cell therapy. Mesenchymal stem cells (MSCs) are multipotent stromal cells that are capable of inducing immunomodulation and regeneration, and have trilineage differentiation when stimulated *in vitro* (1). It is now understood that MSCs have wide-ranging physiological effects, in addition to their regenerative and immunoregulatory capacities (2), which also exhibit anti-inflammation activity (3). Furthermore, MSCs can release a variety of cytokines through paracrine function, or directly interact with immune cells, resulting in immune regulation and inflammation alleviation (4). Based on these prepositional studies, our center is currently working on another randomized and more sophisticated study to verify the therapeutic value of MSC treatment in severe and critically ill patients with COVID-19 pneumonia, with the expectation to promote its potential long-term efficacy and decrease its toxicity.

In consideration of the current dire situation, we would like to share our experience in critical care of an elderly patient who had no existing medical conditions and later transformed into critically ill COVID-19 case. This patient had developed multiple severe virus-induced syndrome and yet successfully recovered after continuous treatments of combined antibiotic therapy and an innovative human umbilical cord mesenchymal stem cell (hUCMSC) intervention. This case highlights our patient's clinical course, including relevant medical history, diagnostic work-up, medical management, and challenges in respiratory function and immunologic function supplement, as well as pulmonary fibrosis repairing.

## Case Description

**Figure 1:**
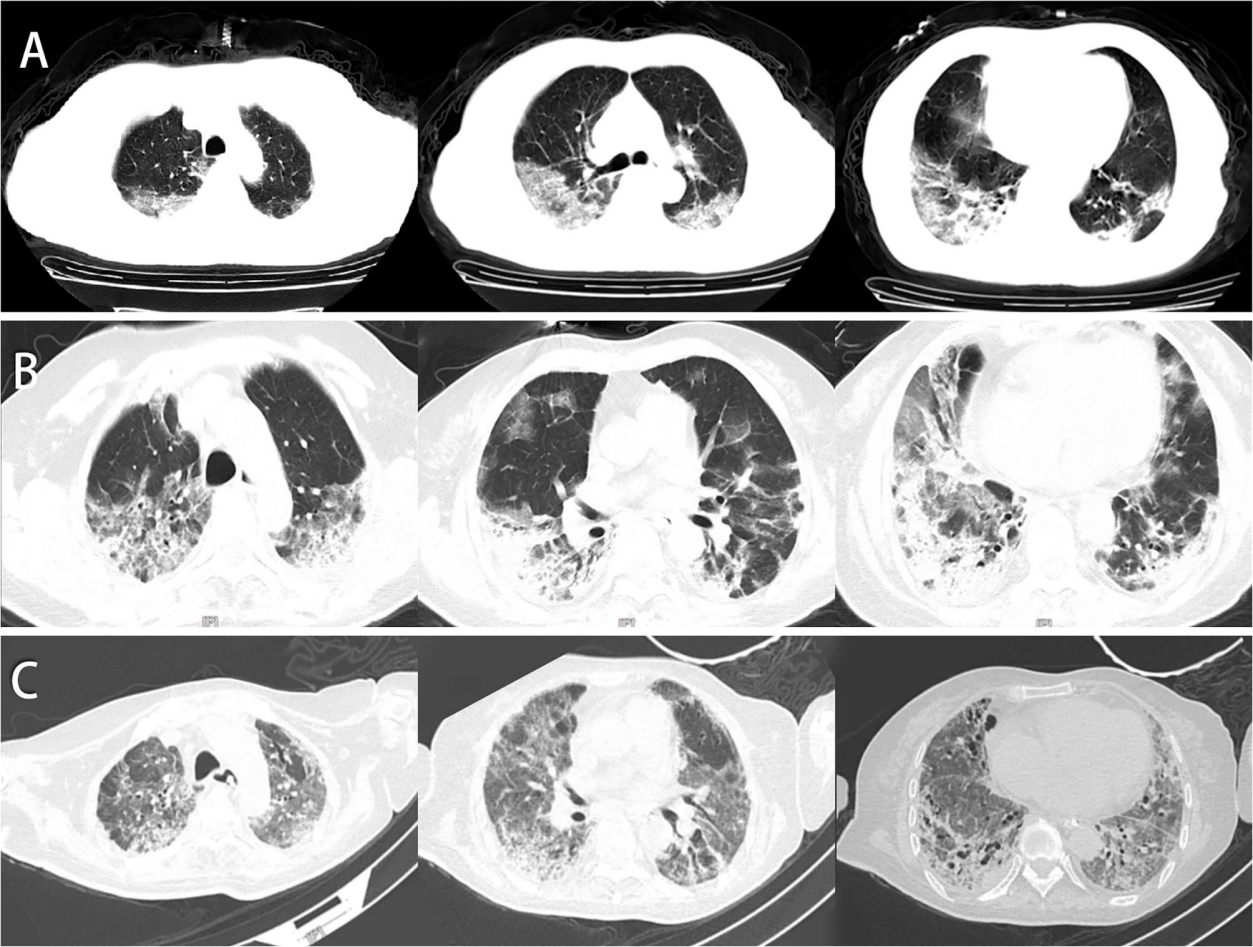
Chest CT of this patient. **(A)** Chest CT was obtained on February 6, 2020 (hospital day 3, illness day 5). Multiple patchy shadows, ground-glass opacity (GGO) under the pleura, streak-like density-increased shadows in the bilateral lungs were observed. **(B)** Chest CT was obtained on February 23, 2020 (day 21). The texture of the trachea and blood vessels in both lungs showed thickening. Both the GGO and patchy shadows increased, and the original was consolidated. **(C)** Chest CT was obtained on April 10, 2020 (day 68). The patchy lesions and consolidations in both lungs were absorbed, and the fiber shadows increased in size partially in the lower lung.

A 66-year-old Chinese woman without a smoking or drinking habit and significant past medical history presented to our institution on February 3, 2020. The chief complaints of this patient were dizziness and vomiting, with diarrhea of watery stool (five times per day) that developed after returning to Changsha from Wuhan 7 days before. On February 2, she visited a local hospital for medical diagnosis and received complete blood count (CBC) test, with a low white blood cell (WBC) count, and chest CT scan showed multiple lesions in the lung. The primary diagnosis was viral pneumonia. Further RT-PCR detection for COVID-19 pathogen was conducted, and the result proved to be positive. Then the patient was transferred to our Institute of Changsha Public Health Center. She had no cough or fever, and showed clear conscience at admission, with vitals: body temperature: 37.3°C, pulse: 72 beats/min, blood pressure: 108/66 mmHg, respiratory rate: 19 breaths/mine, and oxygen saturation: 98% on oxygen inhalation. The patient had thick breath sounds in bilateral lungs. Chest CT examination was performed to evaluate the abnormal breath sounds, which revealed bilateral pneumonia (Figure 1A).

During the first development phase ranging from hospital day 121 (illness days 3–23), the patient presented with dizziness, vomiting, and diarrhea, yet with a little cough and no fever. Our continuous bedside monitoring of her vital signs showed a temperature fluctuation between 37.4 and 38.2°C, except inflammation-induced fever-like symptoms in the initial 2 days (Supplementary Figure 1). Results of CBC test were consistent with those of the viral-infection ramification of high WBC count and low lymphocyte percentage. Chest CT scans from hospital day 20 showed a continuous lesion development in the bilateral lower lobe, with visible fibrosis and necrosis in the lung tissue (Figure 1B). According to the guideline for Diagnosis and Treatment of Pneumonitis with COVID-19 Infection published by the National Health Commission of China (Trial 4th Edition), the patient was treated with antiviral therapy of Arbidol, combined with moxifloxacin, methylprednisolone (40 mg QD), and immunoglobulin (10–20 g QD) from day 3 to 14 for anti-inflammation and reducing overimmunoreaction. A non-invasive mechanical ventilator was used to reduce hypoxia and prevent respiratory muscle fatigue of the patient with oxygen provided through a nasal cannula, after which, high-flow nasal cannula oxygen therapy (HFNC) was provided to her.

On day 22, the patient started to develop hyperthermia symptoms, with the highest body temperature at 38.4°C, indicating failure in antibiotic solution and aggravation of inflammation. In this case, the antibiotic was adjusted to piperacillin/tazobactam to tackle this situation, yet the fever continues. On day 25, meropenem, along with the convalescent plasma (CP) derived from recently recovered donors with neutralizing antibody, substituting previous antibiotics, was introduced to reduce the inflammatory symptoms that continuously occurred. However, the situation got worse, and we reevaluated the patient that she lived in a stage of inflammatory factor storm with high risk to develop into the critically ill type (e.g., multiple organ injury) that required close follow-up. There is an urgent need for safe and alternative therapeutic options to alleviate the storm. Considering the characteristics of hUCMSCs, we speculated that they might be homing to the injured tissues (especially to the lung) and elicit anti-inflammatory function. Thus, our group made a decision to take the hUCMSCs as a therapeutic intervention. The informed consent was obtained by the family member and patient to perform hUCMSC adoptive transfer therapy. The treatment scheme was then discussed, formulated, and approved by the ethics committee of our hospital, with consent forms signed by the family member prior to the treatment. The hUCMSCs were freely derived from the National Engineering Research Center of Human Stem Cells, Changsha, Hunan, China, and belonged to clinical-grade MSCs. The hUCMSCs were prepared on the same day and stored and transported at 4–8°C in strict accordance with standard operating procedures. The total number of infused cells was 1 × 10^6^ cells per kilogram. The allogenic hUCMSCs were administrated intravenously two times (6.4 × 10^7^ cells each time) in the patient on day 28 and 31, at a rate of about 40 drops/min for about 40–50 min. During the treatment, tigecycline, polymyxin B, and voriconazole were added once to resolve the bacterial infections. Following these treatments, the patient temperature stabilized. However, on the midnight of day 29, the patient began to breathe faster, which could not be alleviated by adjusting the ventilator's parameter. The blood oxygen saturation was continuously lower than 86%, and intratracheal intubation was urgently performed to decrease the respiratory distress. However, owing to the continuously deteriorated respiratory function, the patient was provided with the extracorporeal membrane oxygenation (ECMO) support and officially declared a critically ill case with severe pneumonia (mixed type), acute respiratory distress, sepsis, and multiorgan injury (kidney, respiratory system, and heart). Continuous renal replacement therapy (CRRT) was installed for the life support and daptomycin as the temporary critical antibiotic solution. Besides, the planned third infusion on day 34 was suspended owing to the presence of severe mixed coinfections.

During the period from day 31 to 57, the patient was sedated most of the time, accompanied by successive administration of meropenem, or piperacillin/tazobactam, tigecycline, polymyxin B, voriconazole, daptomycin, or linezolid for the antibacteria therapy, as well as continuous thymalfasin and respiratory support. The veno-venous ECMO support was provided through day 30 to 36, but the patient failed to receive endotracheal extubation, and was forced to have a tracheotomy. CBC test showed fluctuated high WBC count, increased hypersensitive C-reactive protein (hCRP) and D-dimer, continuously low lymphocyte counts (Supplementary Table 1), suggesting continued existence of inflammation-inducing sources. Bedside chest X-ray revealed continuous development of ground-glass opacity (GGO) of the whole lung area. All these results indicated a possibly ineffective therapeutic outcome of combined antibiotics and antifungal therapy.

**Figure 2:**
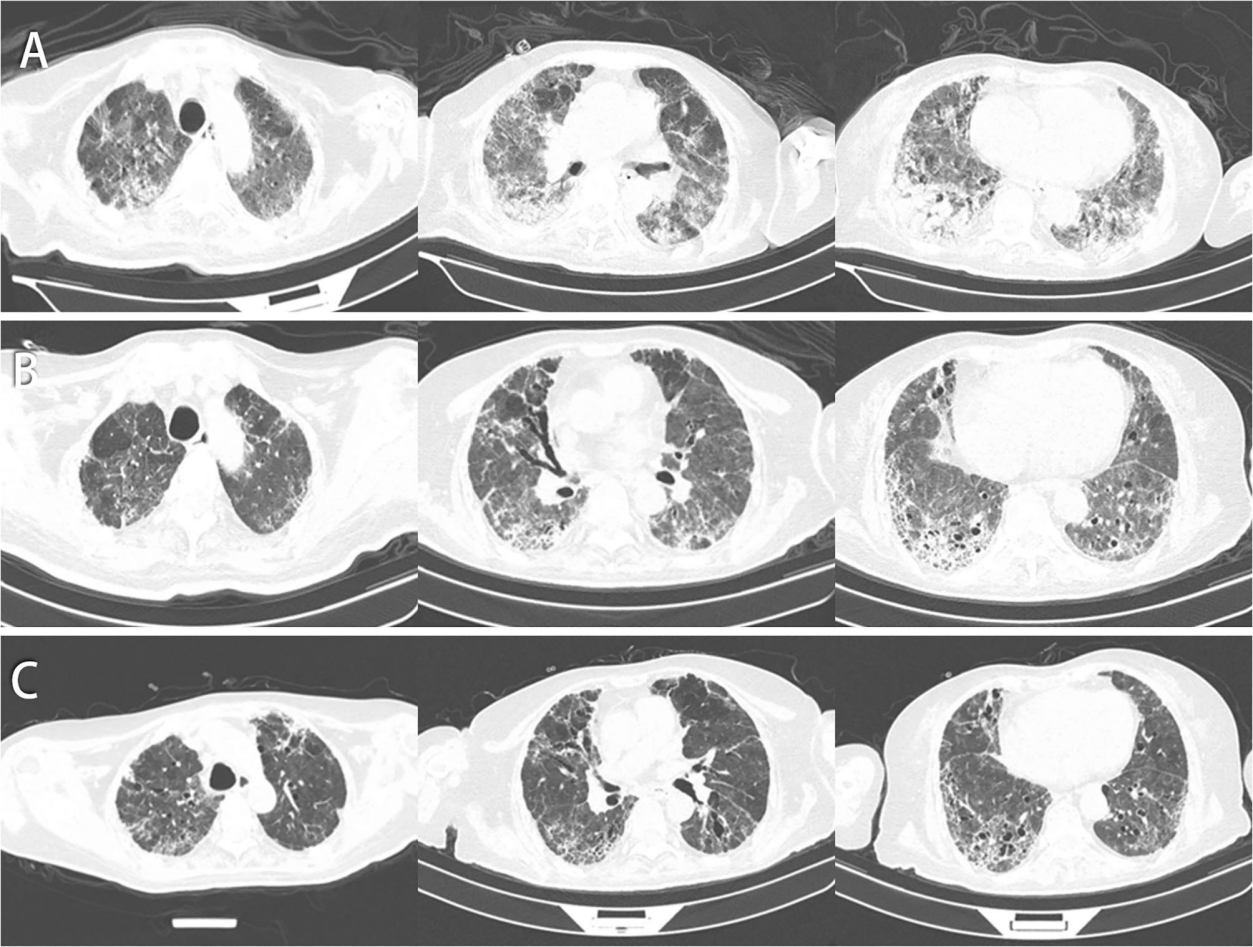
Chest CT of this patient. **(A)** CT images on day 78 indicate that patchy shadows and GGO increased in the right lower lung lobe but decreased in the left upper lung. **(B)** CT images on day 129 indicate that patchy shadows, GGO, and consolidations were absorbed, and some air bronchogram signs and fiber strands were left. **(C)** CT images on day 150 by follow-up indicate that the fiber strands were reduced, and GGO decreased gradually.

On day 58, physical reexamination of the patient showed weakness of breathing and muscles that led to the dependence on a ventilator. Moreover, she had always suffered from a low-to-moderate fever, and comparison of current CT scanning results with the previous showed partial absorption of bilateral patchy lesions in the lungs of the patient, yet accompanied by a great deal of GGO, non-homogeneous density, and air bronchus signs (Figure 1C). Considering the benefits of organ injury recovery caused by inflammatory responses, allogenic hUCMSCs were administrated intravenously for the second round three times (6.5 × 10^7^ cells each time) on days 59, 63, and 66. After the second round of the hUCMSC injection, the patient stayed in the critical care unit (CCU) for continuous monitoring and attentive care for over 20 days. Most of the vital signs and clinical laboratory indexes recovered in the normal range, with certain recovery in the chest CT images as well (Figure 2). On day 87, the patient was transferred out of the CCU for further rehabilitation and finally discharged on hospital day 133. In the subsequent follow-up, the patient can complete proper body exercise, whose activity tolerance was much better than we predicted. For the rehabilitation of pulmonary fibrosis, a third round of hUCMSC administration has been applied on July 7,14, and 21, 2020 (6.5 × 10^7^cells each time). Long-term follow-up is currently ongoing to monitor changes in the lung lesion of the patient (Supplementary Figure 2).

## Observed and Measured Variables

From admission to discharge, clinical symptoms, laboratory tests, and radiological evaluations were recorded and confirmed by a team of trained doctors, especially after receiving hUCMSC infusion. No infusion or allergic reactions, secondary infections, and treatment-related adverse events were found during the observation period. The only controversial hyperthermia and dyspnea occurred on day 29; however, they were not considered to be related to treatment with MSCs because this therapy was just based on the rapid deterioration of the patient's condition, which was characterized by hyperthermia and dyspnea prior to entering the trial. No obvious abnormality was found in the detected concentrations of aspartate aminotransferase, total bilirubin, and creatinine. Collectively, these findings indicated that the use of hUCMSCs was safe and well-tolerated by the patient.

## Discussion and Conclusions

It is commonly known that COVID-19 patients, severe- and critically ill-type cases, in particular, are frequently accompanied by “cytokine storm.” It involves elevated levels of circulating cytokines and immune-cell hyperactivation that can, in turn, lead to lung tissue damage, repair imbalance, and respiratory failure. Cytokine storm is characterized by constitutional symptoms, systemic inflammation, and multiorgan dysfunction that may develop into multiorgan failure if inadequately treated. In our practice, what confuses us is why some COVID-19 patients progress seriously or even die, while some others only show slight clinical symptoms and recover rapidly. Traditional immunology viewpoints cannot well explain the broad spectrum of the disease, and Shi et al. suggested that the course of COVID-19 shall be divided into immune defense-based protective phase and inflammation-driven damaging phase (5). Studies concluded that the first approach in evaluating a patient with cytokine storm is to identify the underlying disorder based on the clinical characteristics and complete workup for laboratory assessment (6). From February 25 (day 22), our case had fever again with progressed dyspnea, accompanied by obvious increase in neutrophil counts, hCRP, ESR, and D-dimer along with chest imaging. Unexpectedly, the same situation happened again on March 25 (day 52). All these findings in our case also supported twice occurrence of cytokine storms. For the long term of disease procession, CP is suitable for early application, which, however, cannot completely reverse the storm and does not benefit critically ill patients with COVID-19 (7). After the second cytokine storm, we adjusted the treatment schedule by not infusing CP any more. With CP and MSC infusion treatment, the patient showed an improved symptom of dyspnea, but got myasthenia and subsequent infection that still required mechanical ventilation for treatment. In addition to descended oxygenation index to below 150, the patchy shadows and GGO increased in the right lower lung conversely. After the second round of MSC infusion, another chest CT in the patient showed that bilateral lesions were mostly absorbed. In terms of clinical symptoms, we observed that she no longer had fever, and showed improved muscle strength of limbs through daily activities, yet with restricted movement and no ability to stand alone. Besides, the patient successfully changed to HFNC and then to the nasal catheter only. The above may be attributed partially to MSCs' regulation of the pulmonary microenvironment (8), through restoring impaired alveolar fluid clearance and alveolar protein permeability (9).

**Figure 3:**
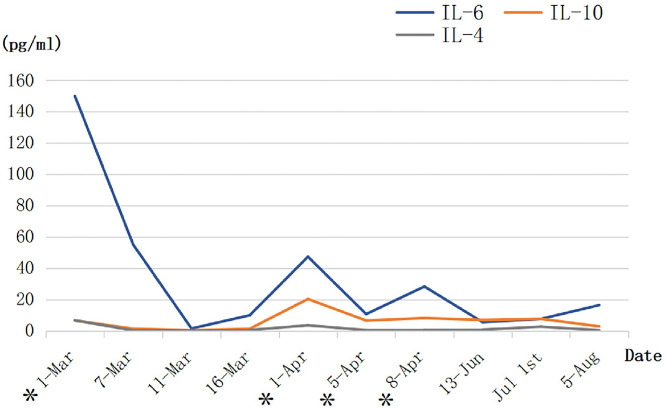
The dynamic changes in the three interleukin levels of the patient during the human umbilical cord mesenchymal stem cell (hUCMSC) treatment period (BD™ Cytometric Bead Array Human Th1/Th2/Th17 Cytokine Kit). *The day of hUCMSC therapy.

Assembling evidence indicates that SARS-CoV-2 spreads mainly through the respiratory tract, either in the form of droplets, breathing secretions, or direct contact. Furthermore, the abundant presence of the ACE2 receptor in lung alveolar epithelial cells promotes the binding of SARS-CoV-2 spike-S-glycoprotein, thus, accelerating viral infection. Critically ill COVID-19 patient data showed the increased levels, to name a few, of IL-2, IL-6, IL-7, IL-10, monocyte chemoattractant protein-1 (MCP-1), and TNF-α (10, 11). MSCs have specific properties to endow them attractive candidates for the therapeutic use of autoimmune inflammatory and tissue damage repairing or tissue homeostasis maintaining. It is well-founded that the primary mechanism by which MSCs exert its therapeutic effects is realized through the secretion of paracrine soluble factors known as secretomes and exosomes (12, 13). This may promote a direct interaction of MSCs with immune cells and perform immune response paracrine modulation by releasing cytokines such as IL-10, IL-1RA, TGF-β, indoleamine 2,3 dioxygenase (IDO), and nitric oxide production. These mechanisms further modulate the proliferation and activation of an anti-inflammatory phenotype of naive and effector T cells, natural killer (NK) cells, and mononuclear cells. Functional modulation of T cells involves inhibition of the Th17 response, induction of regulatory T cells (Treg cells), and shifting from a Th1 to a Th2 cell phenotype (14, 15). It has been documented that MSCs can secrete IL-6 and induce B lymphocytes to produce IgG *in vitro* (16). Additionally, MSCs can prevent neutrophils from apoptosis and degranulation in culture without inhibiting their phagocytic or chemotactic capabilities (17). Based on these knowledge and presumption, an analysis of multiplex cytokine using the patient's whole blood was performed when the patient received hUCMSCs infusion. According to the dynamic changes in IL levels of this patient during the hUCMSC treatment period (Figure 3), IL-6 and IL-10 showed a significant downward trend after infusion and recovered to a certain extent. Similarly, the same changes were observed in WBC, hCRP, and D-dimer as well. These results may indicate that hUCMSCs may reduce inflammation response, and promote the recovery of antiviral T cells and injured tissues when combined with other immunomodulators.

Based on their satisfactory immunomodulatory effect, MSCs have been studied as a promising candidate for treating some inflammatory and immunologic diseases, such as graft-versus-host disease (GvHD), inflammatory bowel disease (IBD), systemic lupus erythematosus (SLE), and ARDS (18–21). As has been clarified, MSCs control both innate and adaptive immune response behavior through release of trophic factors, which are mediated by cell-contact-dependent and -independent mechanisms through the direct cell–cell interaction (22). COVID-19 patients, severe cases in particular, present a consistent decrease in T cells, lower lymphocyte, and higher neutrophil counts in the peripheral blood (23). In comparison, MSCs may increase the generation of Treg cells. Previous validated works have demonstrated its role in inhibiting the proliferation, activation, and maturation of CD19+ B cells, CD4+ Th1 cells, CD8+ T cells, macrophages, monocytes, and neutrophils (19, 24). In the present case, we found that CD4+ T cells, CD8+ T cells, and NK cells all were significantly lower than normal range during a great time of hospitalization (Table 1). These immune cells all play an important role in the immune response of antivirus. It was observed that MSC infusion moderately increased the frequency and count of CD4+ T lymphocytes, suggesting that MSCs most likely affected the T cell subpopulation. In addition, there existed a temporary decline in the count of NK cells, accompanied by decreased counts of WBC and NK cells to a certain extent at different time points after MSC infusions (Supplementary Table 1). Generally speaking, the immunosuppressive effect of MSCs on NK cells is dominant, but substantial data suggests that MSCs can variably affect NK cells and can be affected in turn. The findings support that the controversy depends largely on the ratio of MSC:NK ratios used and sources from which the MSCs and NK cells are isolated (25). On the basis of their abnormal immunophenotype, NK cells have been shown to possess dual roles in the context of COVID-19: an early protective antiviral role and a deteriorative inflammation-promoting role induced by certain subsets (26). Main routes between MSCs and NK cells are considered to include (a) direct cell-to-cell contact, (b) via soluble mediators, and (c) indirectly through modulating the function of other regulatory immune cells like Treg cells (27). In accordance with the aforementioned interpretation, we deem that the improved therapeutic outcome in this patient after MSC infusions in multiple medical conditions can be explained primarily by the development of paracrine factors based on regulation of T lymphocytes, inflammatory mediators, and cytokines, eventually resulting in immunomodulation. The current knowledge herein suggests that MSCs can be regarded as one therapeutic choice possessing a great potential in the management of COVID-19 through targeting NK cells.

**Table 1:** Results of T-lymphocyte cell subsets (BD Multitest™ six-color TBNK).

**Items**	**Normal range (10^**6**^/L)**	**Feb 19**	**Feb 27**	Apr 1^*^	Apr 5^*^	Apr 8^*^	**Apr 21**	**Jul 1**	Jul 14^*^	Jul 21^*^	**Aug 5**
CD3+	1,185–1,901	296	501	127	201	271	358	1,154	1,291	1,098	1,174
CD3+CD8+	404–754	76	175	77	82	119	159	574	610	485	524
CD3+CD4+	361–937	187	325	50	117	151	203	592	644	583	609
CD4/CD8	1.4–2.0	2.46	1.86	0.65	1.43	1.27	1.27	1.03	1.06	1.20	1.16
CD3–CD19+	180–324	39	20	39	70	85	57	46	36	47	58
CD3–CD56+ (NK cell)	175–567	85	158	37	34	72	–	522	552	407	529

* *The day of hUCMSC therapy*.

As supported by the published meta-analysis, quite a number of severe and critically ill COVID-19 patients have received MSC treatment without serious adverse event evidence. The proposed therapy has suggested benefits in reducing mortality and improving pulmonary function in patients with ARDS, as well as in mitigating the physiologic and immunologic responses leading to ARDS (28). In addition, MSCs have powerful antifibrotic effects and may alleviate lung fibrosis. In our case report, according to the follow-up data of chest CT, our patient recovers obviously from pulmonary fibrosis after several times of hUCMSC treatments. Despite no clear evidence to reveal that treatment with stem cells can eradicate coronavirus, preliminary findings are encouraging at this stage, indicating that the patients who are critically ill will be more likely to survive from the infection under this therapy.

## Data Availability Statement

The original contributions presented in the study are included in the article/Supplementary Material, further inquiries can be directed to the corresponding author/s.

## Ethics Statement

The studies involving human participants were reviewed and approved by the ethics committee of First hospital of Changsha. The patients/participants provided their written informed consent to participate in this study. Written informed consent was obtained from the individual(s) for the publication of any potentially identifiable images or data included in this article.

## Author Contributions

All authors contributed to the study conception and design. KH, JH, and AL conceived the proposal for the therapy. KH, JL, XF, and YD organized the clinical study and interpreted the results. QZ and KH prepared the report manuscript. KH, JL, and XF contributed to the clinical observations, material preparation, and image collection. AL, KH, and YZ analyzed and interpreted the X-ray and CT images. XS and LC participated in stem cell production and testing. YD and SW reviewed the manuscript. All authors read and approved the final manuscript.

## Conflict of Interest

The authors declare that the research was conducted in the absence of any commercial or financial relationships that could be construed as a potential conflict of interest.

## References

[1] Dominici M Le Blanc K Mueller I Slaper-Cortenbach I Marini F Krause D. Minimal criteria for defining multipotent mesenchymal stromal cells. The International Society for Cellular Therapy position statement. Cytotherapy. (2006) 8:315–7. 10.1080/14653240600855905 16923606

[2] Levy O Kuai R Siren EMJ Bhere D Milton Y Nissar N. Shattering barriers toward clinically meaningful MSC therapies. Sci Adv. (2020) 6:eaba6884. 10.1126/sciadv.aba6884 32832666 PMC7439491

[3] Bernardo ME Fibbe WE. Mesenchymal stromal cells: sensors and switchers of inflammation. Cell Stem Cell. (2013) 13:392–402. 10.1016/j.stem.2013.09.006 24094322

[4] Prockop DJ Oh JY. Mesenchymal stem/stromal cells (MSCs): role as guardians of inflammation. Mol Ther. (2012) 20:14–20. 10.1038/mt.2011.211 22008910 PMC3255583

[5] Shi Y Wang Y Shao C Huang J Gan J Huang X. COVID-19 infection: the perspectives on immune responses. Cell Death Differ. (2020) 27:1451–4. 10.1038/s41418-020-0530-3 32205856 PMC7091918

[6] Fajgenbaum DC June CH. Cytokine storm. N Engl J Med. (2020) 383:2255–73. 10.1056/NEJMra2026131 33264547 PMC7727315

[7] Omrani AS Zaqout A Baiou A Daghfal J Elkum N Alattar RA. Convalescent plasma for the treatment of patients with severe coronavirus disease 2019: a preliminary report. J Med Virol. (2021) 93:1678–86. 10.1002/jmv.26537 32965715 PMC7537323

[8] Antunes MA Laffey JG Pelosi P Rocco PR. Mesenchymal stem cell trials for pulmonary diseases. J Cell Biochem. (2014) 115:1023–32. 10.1002/jcb.24783 24515922

[9] Loy H Kuok DIT Hui KPY Choi MHL Yuen W Nicholls JM. Therapeutic implications of human umbilical cord mesenchymal stromal cells in attenuating influenza A(H5N1) virus-associated acute lung injury. J Infect Dis. (2019) 219:186–96. 10.1093/infdis/jiy478 30085072 PMC6306016

[10] Huang C Wang Y Li X Ren L Zhao J Hu Y. Clinical features of patients infected with 2019 novel coronavirus in Wuhan, China. Lancet. (2020) 395:497–506. 10.1016/S0140-6736(20)30183-5 31986264 PMC7159299

[11] Liuzzo G Patrono C. COVID 19: in the eye of the cytokine storm. Eur Heart J. (2021) 42:150–1. 10.1093/eurheartj/ehaa1005 33462598 PMC7799110

[12] Lee JW Gupta N Serikov V Matthay MA. Potential application of mesenchymal stem cells in acute lung injury. Expert Opin Biol Ther. (2009) 9:1259–70. 10.1517/14712590903213651 19691441 PMC2852252

[13] Askenase PW. COVID-19 therapy with mesenchymal stromal cells (MSC) and convalescent plasma must consider exosome involvement: do the exosomes in convalescent plasma antagonize the weak immune antibodies? J Extracell Vesicles. (2020) 10:e12004. 10.1002/jev2.12004 33304473 PMC7710130

[14] Pittenger MF Discher DE Peault BM Phinney DG Hare JM Caplan AI. Mesenchymal stem cell perspective: cell biology to clinical progress. NPJ Regen Med. (2019) 4:22. 10.1038/s41536-019-0083-6 31815001 PMC6889290

[15] Chow L Johnson V Impastato R Coy J Strumpf A Dow S. Antibacterial activity of human mesenchymal stem cells mediated directly by constitutively secreted factors and indirectly by activation of innate immune effector cells. Stem Cells Transl Med. (2020) 9:235–49. 10.1002/sctm.19-0092 31702119 PMC6988770

[16] Rasmusson I Le Blanc K Sundberg B Ringden O. Mesenchymal stem cells stimulate antibody secretion in human B cells. Scand J Immunol. (2007) 65:336–43. 10.1111/j.1365-3083.2007.01905.x 17386024

[17] Raffaghello L Bianchi G Bertolotto M Montecucco F Busca A Dallegri F. Human mesenchymal stem cells inhibit neutrophil apoptosis: a model for neutrophil preservation in the bone marrow niche. Stem Cells. (2008) 26:151–62. 10.1634/stemcells.2007-0416 17932421

[18] Wilson JG Liu KD Zhuo H Caballero L McMillan M Fang X. Mesenchymal stem (stromal) cells for treatment of ARDS: a phase 1 clinical trial. Lancet Respir Med. (2015) 3:24–32. 10.1016/S2213-2600(14)70291-7 25529339 PMC4297579

[19] Gao L Zhang Y Hu B Liu J Kong P Lou S. Phase II multicenter, randomized, double-blind controlled study of efficacy and safety of umbilical cord-derived mesenchymal stromal cells in the prophylaxis of chronic graft-versus-host disease after HLA-haploidentical stem-cell transplantation. J Clin Oncol. (2016) 34:2843–50. 10.1200/JCO.2015.65.3642 27400949

[20] Hosseini-Asl SK Mehrabani D Karimi-Busheri F. Therapeutic effect of mesenchymal stem cells in ulcerative colitis: a review on achievements and challenges. J Clin Med. (2020) 9:3922. 10.3390/jcm9123922 33287220 PMC7761671

[21] Wang D Li J Zhang Y Zhang M Chen J Li X. Umbilical cord mesenchymal stem cell transplantation in active and refractory systemic lupus erythematosus: a multicenter clinical study. Arthritis Res Ther. (2014) 16:R79. 10.1186/ar4520x 24661633 PMC4060570

[22] Corcione A Benvenuto F Ferretti E Giunti D Cappiello V Cazzanti F. Human mesenchymal stem cells modulate B-cell functions. Blood. (2006) 107:367–72. 10.1182/blood-2005-07-2657 16141348

[23] Diao B Wang C Tan Y Chen X Liu Y Ning L. Reduction and functional exhaustion of T cells in patients with coronavirus disease 2019 (COVID-19). Front Immunol. (2020) 11:827. 10.3389/fimmu.2020.00827 32425950 PMC7205903

[24] Leyendecker A Jr. Pinheiro CCG Amano MT Bueno DF. The use of human mesenchymal stem cells as therapeutic agents for the *in vivo* treatment of immune-related diseases: a systematic review. Front Immunol. (2018) 9:2056. 10.3389/fimmu.2018.02056 30254638 PMC6141714

[25] Thomas H Jager M Mauel K Brandau S Lask S Flohe SB. Interaction with mesenchymal stem cells provokes natural killer cells for enhanced IL-12/IL-18-induced interferon-gamma secretion. Mediators Inflamm. (2014) 2014:143463. 10.1155/2014/143463 24876666 PMC4021755

[26] Alrubayyi A. NK cells in COVID-19: protectors or opponents? Nat Rev Immunol. (2020) 20:520. 10.1038/s41577-020-0408-0 32732951 PMC7391014

[27] Moloudizargari M Govahi A Fallah M Rezvanfar MA Asghari MH Abdollahi M. The mechanisms of cellular crosstalk between mesenchymal stem cells and natural killer cells: therapeutic implications. J Cell Physiol. (2021) 236:2413–29. 10.1002/jcp.30038 32892356

[28] Qu W Wang Z Hare JM Bu G Mallea JM Pascual JM. Cell-based therapy to reduce mortality from COVID-19: systematic review and meta-analysis of human studies on acute respiratory distress syndrome. Stem Cells Transl Med. (2020) 9:1007–22. 10.1002/sctm.20-0146 32472653 PMC7300743

