# Supplementary material for: Case Report: Human Umbilical Cord Mesenchymal Stem Cells as a Therapeutic Intervention for a Critically Ill COVID-19 Patient
Source: Front Med (Lausanne). 2021 Jul 8;8:691329. doi: 10.3389/fmed.2021.691329 (PMC8298026; doi:10.3389/fmed.2021.691329)
Supplement: Supplementary file 1 [file Table_1.DOCX]

**Table S1** Clinical Laboratory Results

| **Measurements** | **Reference**  **range** | **Feb3** | **Feb25** | **Mar1**^∗^ | **Mar4**^∗^ | **Mar7** | **Mar25** | **Apr1**^∗^ | **Apr5**^∗^ | **Apr8**^∗^ | **Apr13** | **Jul1** |
| --- | --- | --- | --- | --- | --- | --- | --- | --- | --- | --- | --- | --- |
| White-cell count (10^9^/L) | 4-10 | 2.79 | 6.93 | 7.85 | 5.99 | 4.82 | 18.74 | 4.92 | 2.73 | 2.39 | 6.8 | 8.11 |
| Red-cell count(10^9^/L) | 3.5-5.5 | 4.32 | 3.34 | 3.69 | 2.67 | 2.59 | 3.38 | 2.67 | 2.91 | 2.89 | 3.08 | 4.32 |
| neutrophil count | 2-7 | 1.95 | 5.34 | 6.03 | 6.23 | 3.41 | 17.36 | 4.42 | 2.3 | 1.68 | 5.71 | 5.57 |
| lymphocyte count (10^9^/L) | 0.8-4 | 0.62 | 0.86 | 0.94 | 0.31 | 0.92 | 0.81 | 0.35 | 0.36 | 0.54 | 0.54 | 1.86 |
| Platelet count (10^9^/L) | 100-300 | 117 | 210 | 242 | 199 | 141 | 220 | 217 | 177 | 193 | 286 | 215 |
| Hemoglobin (g/L) | 110-160 | 125 | 99 | 108 | 80 | 76 | 105 | 84 | 92 | 92 | 99 | 132 |
| Alanine aminotransferase (U/L) | 0-42 | 16.2 | 40.0 | 35.6 | 29.9 | 20 | 49.1 | 5.5 | 15.6 | 26.3 | 17.7 | 37.7 |
| Aspartate aminotransferase (U/L) | 0-37 | 33.2 | 33.7 | 31.2 | 29.8 | 24.1 | 79.1 | 53.9 | 30.8 | 34.7 | 23.4 | 26.9 |
| Albumin (g/L) | 35-55 | 35.36 | 40.1 | 28.3 | 30.8 | 26.7 | 32.3 | 29.3 | 29.9 | 29 | 33.8 | 36.2 |
| Creatinine (μmol/L) | 19.8-87.1 | 26.84 | 37.0 | 37.7 | 42 | 48.1 | 114.4 | 112.6 | 45.5 | 26.4 | 36.1 | 43.7 |
| Lactate dehydrogenase (U/L) | 80-245 | 184.9 | 327.0 | 271.5 | 301.9 | 254.2 | 515.3 | 479 | 426 | 378 | 299 | 232 |
| ESR | 0-15 | 43 | 125 | 123 | 122 | 77 | 109 | 120 | - | 80 | - | 34 |
| HC-reactive protein (mg/L) | 0-8 | 18.73 | ＞80.3 | ＞80.3 | 42.7 | ＞80.3 | 50.4 | 76 | 24.8 | 21.3 | 31.3 | 6.05 |
| Procalcitonin (ng/ml) | 0-0.05 | 0.056 | <0.05 | <0.05 | 9.32 | 0.34 | 0.67 | 0.385 | 0.6 | 0.15 | 0.17 | - |
| PaO_2_/FiO_2_(mmHg) | 400-500 | 363.3 | 176.8 | 107.7 | 162 | 265.7 | 141.8 | 201.5 | 220 | 235 | 296.5 | - |
| D-Dimer (μg/ml) | 0-1 | 0.16 | 9.18 | - | 13.73 | 10.91 | 1.49 | 2.42 | 1.52 | - | 1.028 | 0.88 |
| Nucleic acid detection | Negative |  |  | Positive |  | Negative |  |  |  |  |  |  |

hUCMSC = human umbilical cord mesenchymal stem cell.

∗Indicates the day of hUCMSCs therapy.

_Indicates the condition of ECMO.
