# Supplementary material for: Case Report: Human Umbilical Cord Mesenchymal Stem Cells as a Therapeutic Intervention for a Critically Ill COVID-19 Patient
Source: Front Med (Lausanne). 2021 Jul 8;8:691329. doi: 10.3389/fmed.2021.691329 (PMC8298026; doi:10.3389/fmed.2021.691329)
Supplement: Supplementary file 2 [file Data_Sheet_1.DOC]

Supplementary Material

**Legend**

**Figure S1** Clinical symptoms and main therapies from February 3rd to March 3rd, 2020.

**Figure S2** Follow-up CT images on August 5,2020 and January 6,2021. The fiber strands and GGO were nearly absorbed.

**Table S1** Clinical Laboratory Results
