# Supplementary figures and images for: Case Report: Human Umbilical Cord Mesenchymal Stem Cells as a Therapeutic Intervention for a Critically Ill COVID-19 Patient
Source: Front Med (Lausanne). 2021 Jul 8;8:691329. doi: 10.3389/fmed.2021.691329 (PMC8298026; doi:10.3389/fmed.2021.691329)

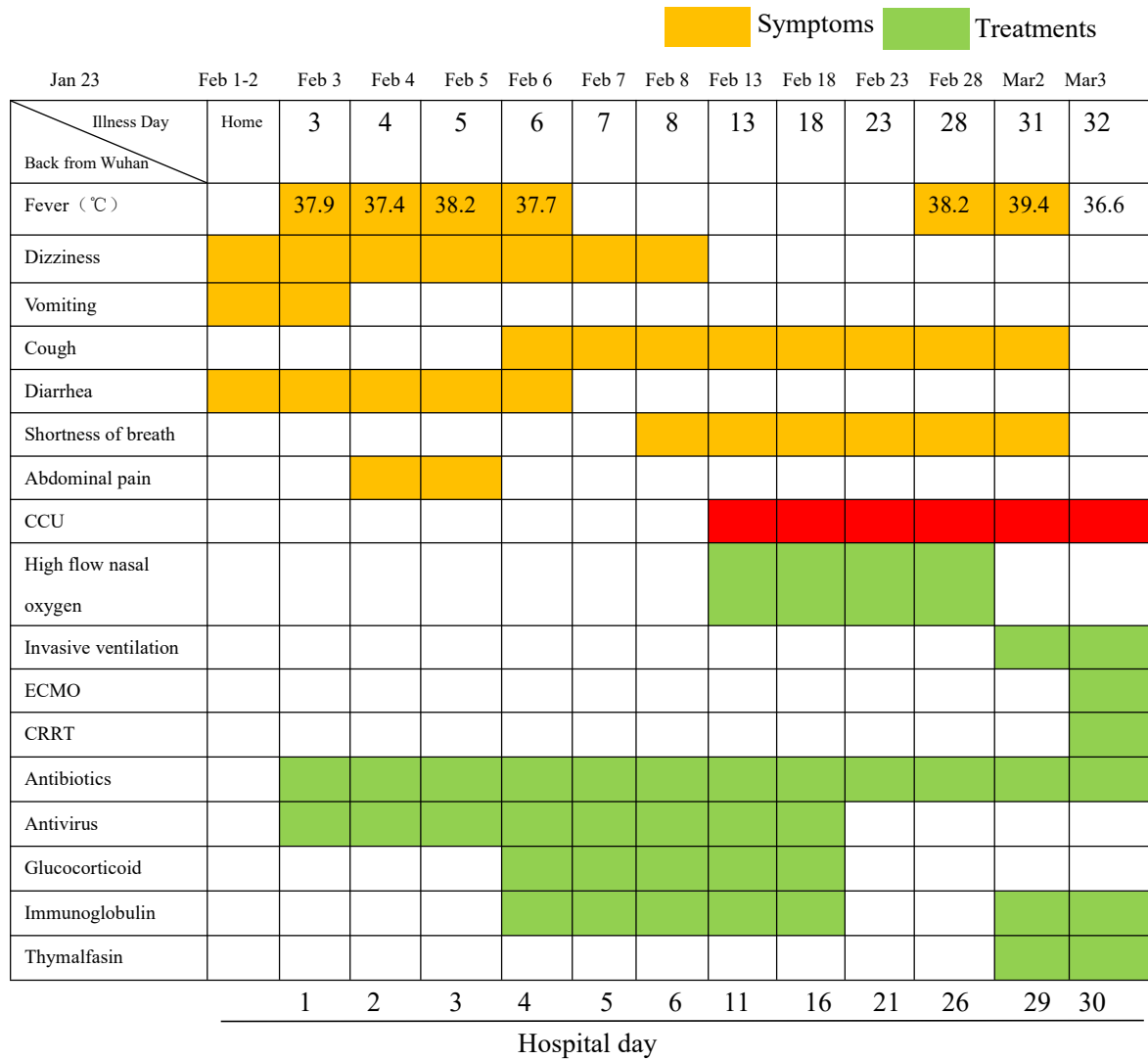

Fig.2 Clinical symptoms and main therapies from Feb 3rd to Mar 3rd, 2020.

Supplement: Supplementary file 3 [file Data_Sheet_2.PDF]

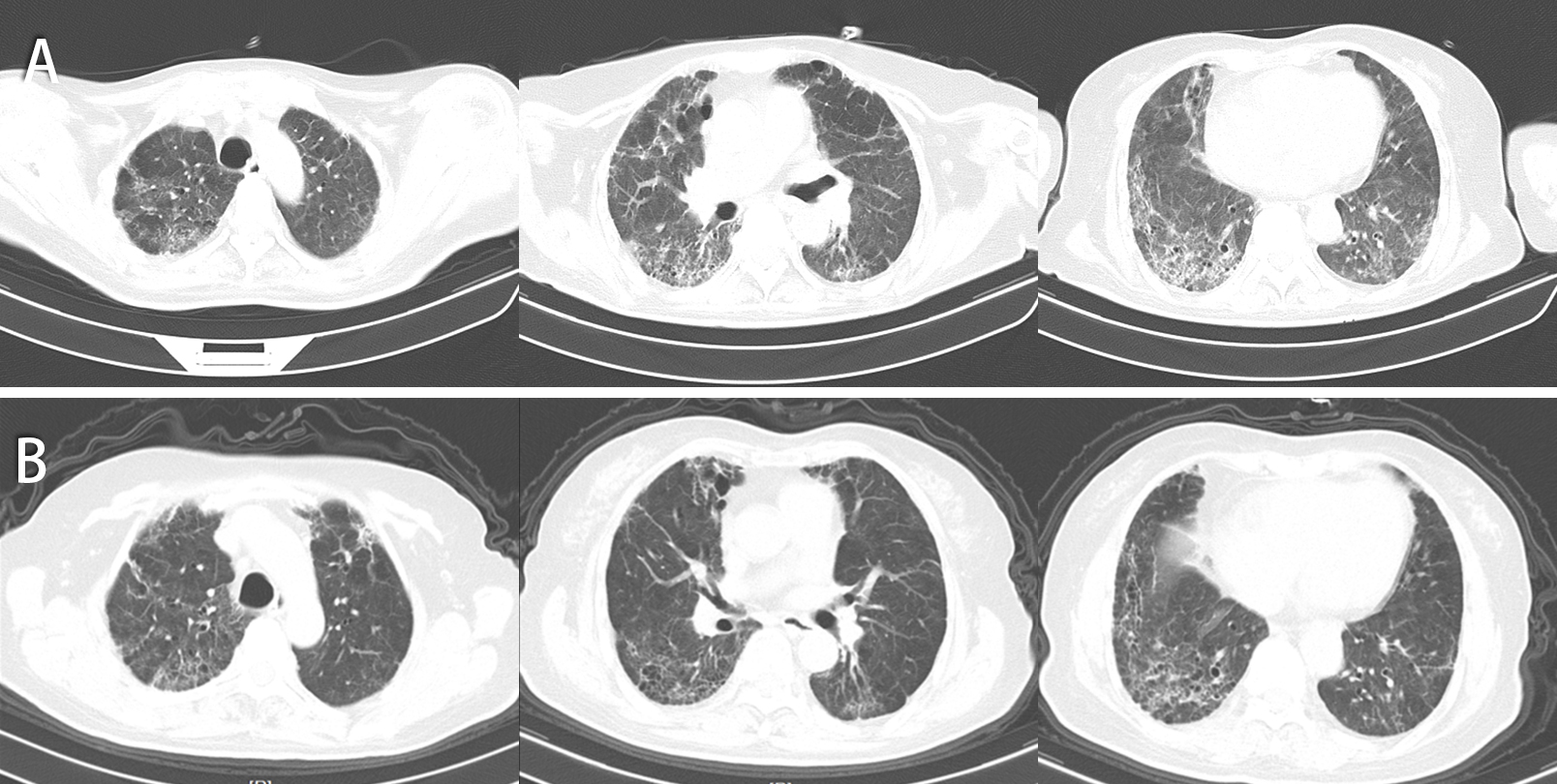

Supplement: Supplementary file 4 [file Image_1.JPEG]
